# Sugar Composition Analysis of Fuzi Polysaccharides by HPLC-MS^n^ and Their Protective Effects on Schwann Cells Exposed to High Glucose

**DOI:** 10.3390/molecules21111496

**Published:** 2016-11-09

**Authors:** Bei-Bei Wang, Jia-Li Wang, Jiang Yuan, Qing-Hua Quan, Rui-Fang Ji, Peng Tan, Jing Han, Yong-Gang Liu

**Affiliations:** 1 School of Chinese Materia Medica, Beijing University of Chinese Medicine, Wangjing Zhonghuan Road No. 6 School Range, Chaoyang District, Beijing 100102, China; wbb20131681@163.com (B.-B.W.); wangjl120@163.com (J.-L.W.); Bucmyj2015@163.com (J.Y.); 18810689952@163.com (Q.-H.Q.); 17801164351@163.com (R.-F.J.); tanpengtcm@163.com (P.T.); 2 Beijing Chinese Medicine Research Institute, Beijing University of Chinese Medicine, North Third Ring Road No. 11 School Range, Chaoyang District, Beijing 100029, China

**Keywords:** Fuzi polysaccharides, HPLC-ESI-MS^n^, monosaccharide composition, Schwann cells, antioxidant, AMPK-PGC-1α pathway

## Abstract

Fuzi has been used to treat diabetic complications for many years in china. In a previous study, we have shown that Fuzi aqueous extract can attenuate Diabetic peripheral neuropathy (DPN) in rats and protect Schwann cells from injury. Thus, the protective effect of Fuzi polysaccharides (FPS) on high glucose-induced SCs and the preliminary mechanism were investigated. Firstly, the FPS were obtained and their monose composition was analyzed by the combination of pre-column derivatization and high performance liquid chromatography coupled with electrospray ionization multi-tandem mass spectrometry (HPLC/ESI-MS^n^). The results witnessed the efficiency of this method and seven monosaccharides were tentatively identified, among which fucose was first reported. Simultaneously, *m*/*z* 215 can be considered as diagnostic ions to confirm the number of monosaccharides. Next, high glucose-induced SC model was applied and divided into model group, treated group of FPS, normal and osmotic control group. After treatment for 48 h, the data showed FPS could significantly decrease the intracellular ROS and apoptosis, which were determined by the corresponding fluorescent probes. Then, the expression of oxidative stress-related proteins in SCs were measured by Western blot. Furthermore, the protein tests found that FPS markedly up-regulated superoxide dismutase (SOD), catalase (CAT) and peroxisome proliferator-activated receptor gamma coactivator 1-alpha (PGC-1α) protein level, but down-regulated NADPH oxidase-1 (Nox1) protein level. Moreover, FPS could also increase AMP-activated protein kinase (AMPK) activation significantly. Hence, we preliminary deduced that AMPK-PGC-1α pathway may play an important role in the protective effect of FPS against high glucose-induced cell damage.

## 1. Introduction

Diabetic peripheral neuropathy (DPN), the commonest diabetes complication, may affect 30%–90% diabetics and forms a leading cause of foot ulceration and lower limb amputation in patients with DM and consequently burdens their quality of life [1]. Numerous mechanisms can result in DPN, including the activation of polyol pathway flux and protein kinase C, the stack of advanced glycation end products and the overproduction of oxygen free radicals, etc. Thus, it is difficult to find drugs to ameliorate the multiple symptoms of DPN due to the complex mechanism. However, in 2001, a uniform pathological mechanism about diabetic complications was proposed that the increased oxidative stress might intimately associate with nerve dysfunction and regenerative capacity diminishment [2,3]. Subsequently, antioxidants became a hot spot in the management of DPN and drug target [4,5,6].

Schwann cells (SCs), a constituent of myelin cells, play a crucial role in the peripheral nervous system by providing myelin sheath; maintaining, nurturing, and restoring axons and neurons; and producing neurotrophic factors such as nerve growth factor (NGF), and fibroblast growth factor (FGF) [7]. Therefore, the injury of SCs can often cause depressed nerve conduction velocity, atrophic axon, and the impaired regeneration ability of the axon. It has been reported that when SCs are exposed to high glucose concentration, the cell damage could be triggered via induced oxidative stress, activated mitochondrial pathway and promoted apoptosis [8,9]. Hence, high glucose stimulated SCs have been developed as a valuable model for exploring novel therapeutic approaches and further mechanisms of DPN [10,11,12,13,14].

Fuzi, the lateral root of *Aconitum carmichaeli* Debx, was listed in materia medica classics, Shen Nong Ben Cao Jing (Shennong’s Herbal) from 2000 years ago and its medicinal effect against diabetes can be traced back to Shang Han Lun (Treatise on Cold-Induced Diseases) [15]. Besides, modern clinical studies also proved that prescriptions with Fuzi have unexpected effect on treatment of DPN [16,17]. Due to the complex component of the TCM (traditional Chinese medicine) combination formulas, it is not sufficient to validate the effectivity of Fuzi for DPN. In a previous study [18], we have demonstrated that the aqueous extract of Fuzi could ameliorate the symptom of rats with DPN, reduce hydrogen peroxide and superoxide anion induced by high glucose in Schwann cells. In this paper, further exploration of the main functional components of Fuzi for improving the oxidation damages in SCs and the underlying mechanisms were given.

In accordance with our screen, polysaccharides of Fuzi were found to be the active ingredient for cytoprotective effect on SCs. FPS were extracted and purified and its monosaccharide composition was identified using HPLC-MS^n^ for the first time. Then protective effects of FPS on oxidative injury were evaluated with high glucose-induced SCs. Additionally, oxidative stress-related signaling pathways were analyzed by Western blot method for validating the potential molecular mechanism.

## 2. Results

### 2.1. The MS Analysis of FPS

Monosaccharide composition analysis of polysaccharides is of fundamental importance for the research on polysaccharide structure and its characteristics. Pre- or post-column derivatization methods are commonly used in chromatography separation and detection in that monosaccharides have strong polar, similar structure, a lack of optical activity. The reagent PMP (1-Phenyl-3-methyl-5-pyrazolone), one of the common labels that can react with reducing carbohydrate under mild condition, started to be applied in analysis saccharides in 1989 by the Honda [19]. Then pre-column derivatization with PMP method started to be applied in analysis saccharides by HPLC-MS in 1998 [20]. However, with salt formation in traditional derivative reaction, the desalinization treatment has to be done before mass spectrometry. Over the past decade, an improved PMP derivatization method had been developed, which could be applied directly to mass spectrometry [21]. In this study, the improved method was applied to analyze FPS for the first time by HPLC-MS^n^. 

#### 2.1.1. The Fragmentation Regulations of PMP-Labeled Monosaccharides

**Figure 1:**
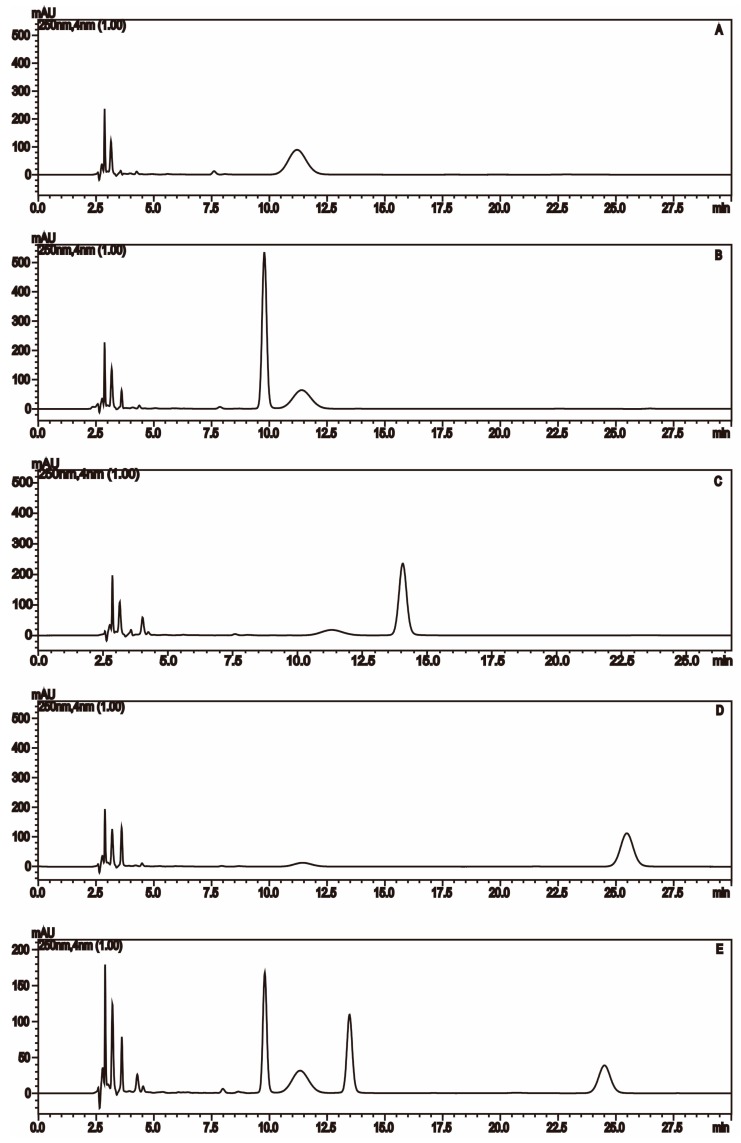
Chromatogram of three double PMP-labeled monosaccharides: (**A**) Blank control; (**B**) PMP-labeled mannose; (**C**) PMP-labeled rhamnose; (**D**) PMP-labeled glucose; and (**E**) three mixed PMP-labeled monosaccharides.

**Figure 2:**
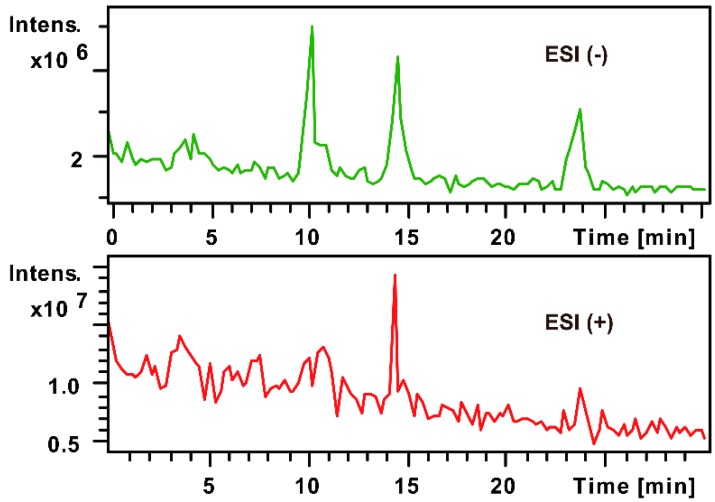
Positive and negative total ion current chromatogram (TIC) of three double PMP-labeled monosaccharides.

**Figure 3:**
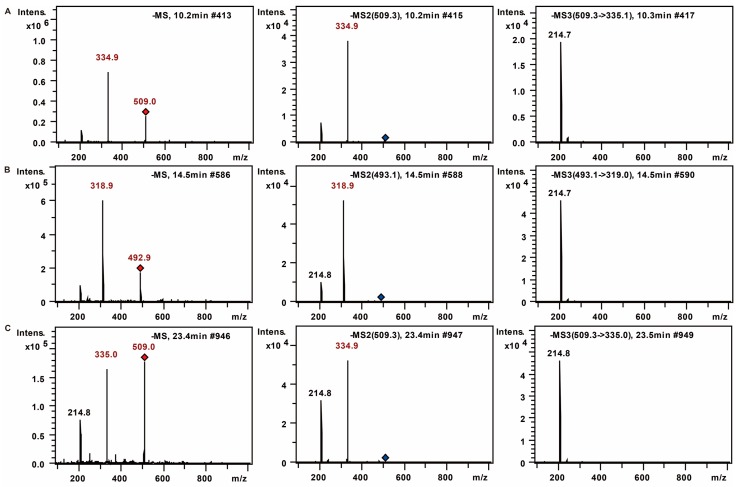
MS (**left**); MS^2^ (**middle**); and MS^3^ (**right**) spectra of PMP-labeled: mannose (**A**); rhamnose (**B**); and Glucose (**C**).

**Figure 4:**
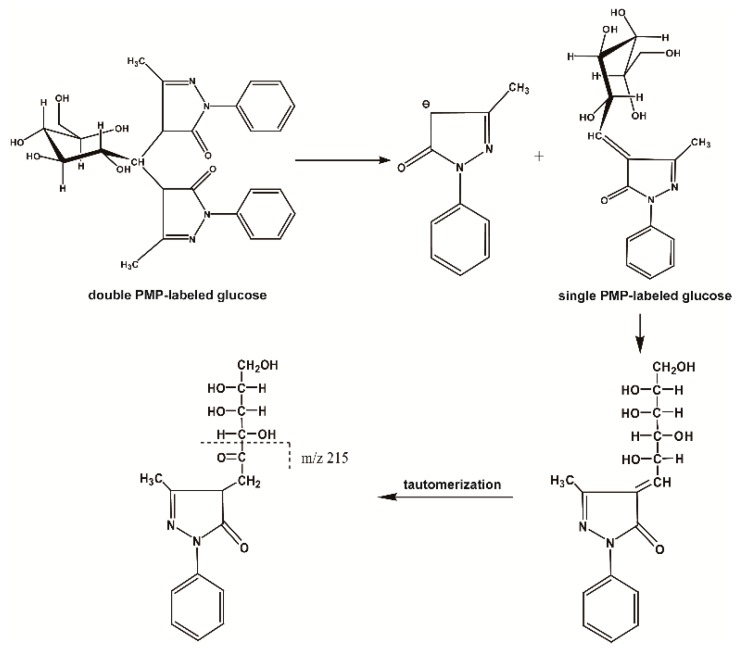
Fragmentation pathway of double PMP-labeled monosaccharides in ESI-MS^n^.

Firstly, through the comparison between the blank and PMP-labeled group, it can be sure that derivative reaction was completed successfully (see Figure 1). Compared with positive-ion, negative-ion mode has a weaker interference of background, although both ion modes have high response (see Figure 2). The [M − H]^−^, [M − PMP − H]^−^ were obtained easily in MS. Then, in MS^3^, a characteristic fragment ions *m*/*z* 215 were detected, which could be used to confirm the number of monosaccharide with negative ion mode (see Figure 3). The fragmentation pathway is shown in Figure 4.

#### 2.1.2. Monosaccharide Composition Analysis of the Hydrolyzed FPS

**Figure 5:**
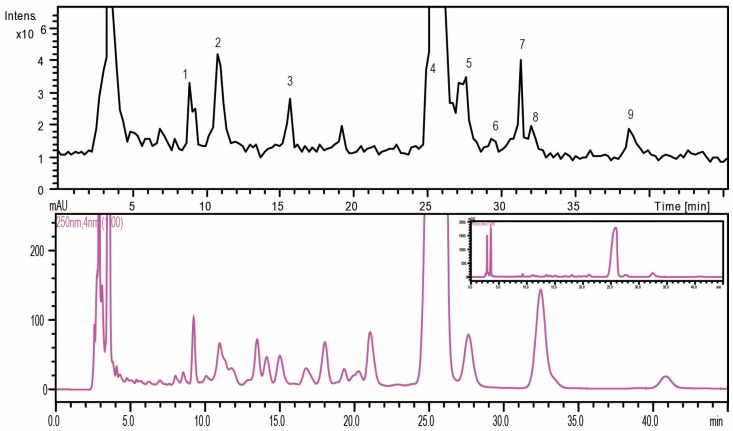
The total ion chromatogram and chromatogram of PMP-labeled monosaccharide composition of polysaccharides.

Total ion chromatograms (TIC) of the separated PMP-labeled monosaccharides were detected simultaneously (Figure 5). In our study, nine PMP-labeled monosaccharides were separated, in which seven were identified according to fragmentation characterization of PMP-labeled monosaccharides and references [22,23,24] (Table 1).

Peaks 2, 3, and 5 could be undoubtedly identified as PMP-labeled mannose, rhamnose, and glucose according to the retention times and MS data of standard sample. The number of monosaccharide was determined through the characteristic fragment ions *m*/*z* 215. Further, the molecular weight was obtained by the fragments [M − H]^−^, [M − PMP − H]^−^. Based on the retention times on the C18 columns, the monosaccharide composition was tentatively identified.

To recap, FPS was composed of mannose, glucose, rhamnose, galactose, xylose, fucose and arabinose, which mainly were glucose.

### 2.2. Evaluation of the Effects of FPS on Reactive Oxygen Species in High Glucose-Stimulated RSC96 Cells

**Figure 6:**
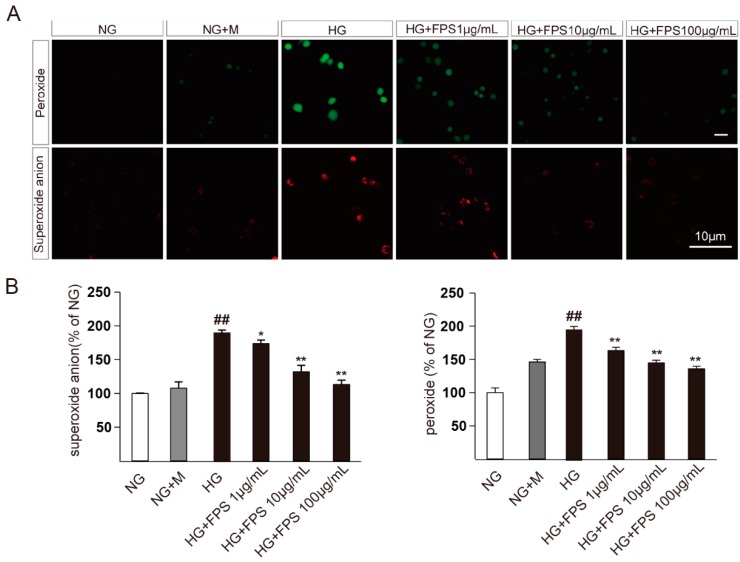
The effects of FPS on antioxidant in injured RSC96 Cells: (**A**) RSC96 cells were stained with DCFH_2_DA and DHE; and (**B**) the quantized data of peroxide and superoxide anion levels were expressed by mean ± S.D. (*n* = 3). ## or ** *p* < 0.01; * *p* < 0.05; “##”, HG vs. NG; “*” or “**”, HG vs. HG + FPS. The ordinate is a percentage relative to NG group.

The antioxidative effects of FPS on high glucose-stimulated RSC96 cells cultivated in different treatment for 48 h are shown in Figure 6. The levels of peroxide and superoxide anions in NG + M group increased slightly compared to the NG group. However, the HG group increased ROS significantly, which revealed that the sugar concentration was the main causes of oxidative injury in RSC96 cells. Moreover, the result displayed that all doses of FPS could dramatically down-regulate peroxide and superoxide anion levels in a dose-dependent manner (*p* < 0.01). Therefore, FPS has an inhibitory effect on hyperglycemia-induced ROS in RSC96 Cells.

### 2.3. Evaluation of the Effects of FPS on Apoptosis in High Glucose-Stimulated RSC96 Cells

**Figure 7:**
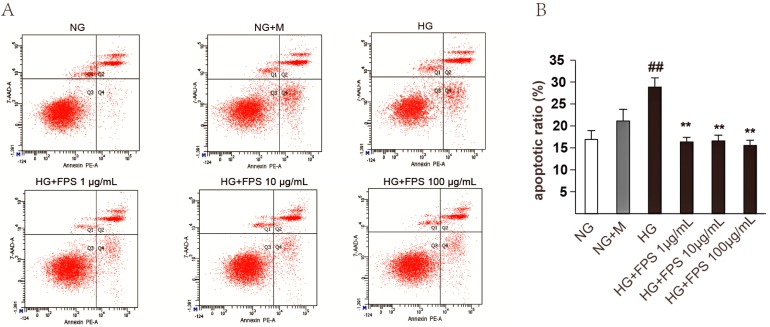
The effects of FPS on antiapoptotic in injured RSC96 Cells: (**A**) Annexin V-PE and 7-AAD staining was represented by flow cytometry. Q1: necrotic; Q2: late apoptotic cells; Q3: normal viable cells; Q4: early stage apoptotic cells; (**B**) Apoptosis rate = early apoptosis + late apoptosis, the date were expressed by mean ± S.D. (*n* = 3). ## or ** *p* < 0.01; “##”, HG vs. NG; “**”, HG vs. HG + FPS.

The antiapoptotic effect of FPS on high glucose-stimulated RSC96 cells was measured by flow cytometry using annexin V-PE and 7-AAD staining. As can be seen in Figure 7, the apoptotic ratio had no obvious change between normal control and osmotic control group. Unsurprisingly, significant increases were observed in the apoptotic ratio of RSC96 cells stimulated by high glucose. Compared with HG group, the apoptotic ratio was restored (*p* < 0.01) in medicated group of FPS, whereas the curative effects did not show significant changes as the concentration of FPS rose.

### 2.4. Evaluation of the Effects of on the Expression of Antioxidant Enzymes and Nox1

**Figure 8:**
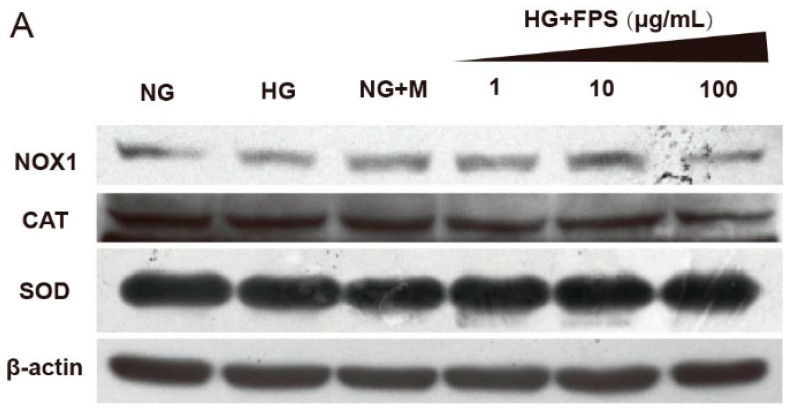

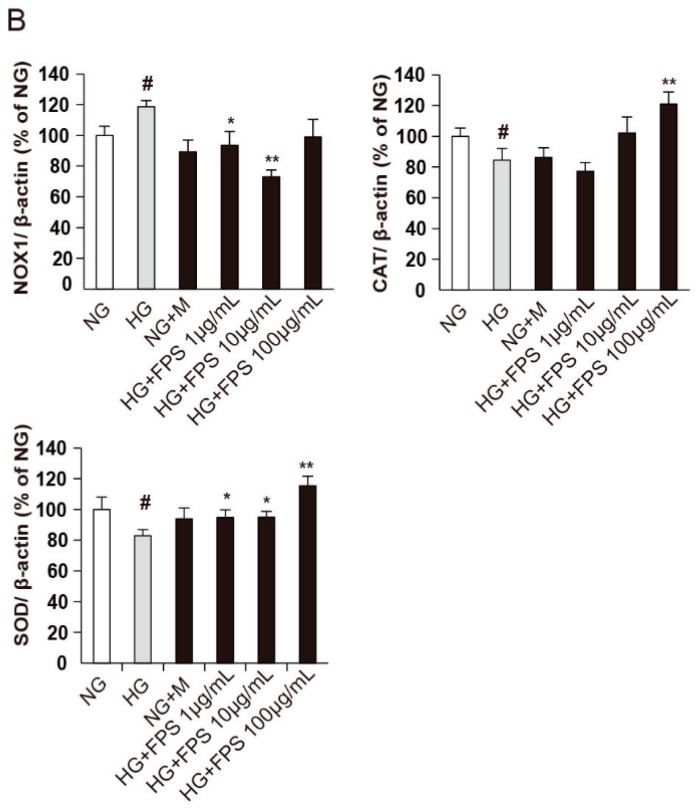
The effects of FPS on the expression of antioxidant enzymes and Nox1: (**A**) The protein expression levels were assayed by Western blotting; (**B**) The statistical data of protein were expressed by mean ± S.D. (*n* = 3). ** *p* < 0.01; # or * *p* < 0.05; “#”, HG vs. NG; “*” or “**”, HG vs. HG + FPS. The ordinate is the percentage relative to NG group.

To clarify the mechanism underlying the inhibitory effect of FPS on ROS production, we detected the expression of enzymes including SOD, CAT, and Nox1 which were directly related to the formation and removal of ROS [25,26]. Compared with NG, the protein expression of SOD and CAT were reduced in HG group (*p* < 0.05). Moreover, the Nox1 protein level was increased in HG group (*p* < 0.05). As expected, FPS could suppress the expression of Nox1 at different concentrations (*p* < 0.05 or *p* < 0.01) and, simultaneously, FPS upregulated CAT and SOD protein expression (*p* < 0.05 or *p* < 0.01) (Figure 8).

### 2.5. The Effects of FPS on AMPK-PGC-1α Pathway

Then, we further investigated PGC-1α, a transcriptional coactivator required for the induction of many ROS-detoxifying enzymes and a master regulator of mitochondrial biogenesis and AMPK, an essential upstream mediator of PGC-1α [27,28,29]. The results showed that HG attenuated both the ratio of p-AMPK/AMPK) and the expression of PGC-1α (*p* < 0.05 or *p* < 0.01). Moreover, FPS increased levels of p-AMPK and PGC-1α in a dose-dependent manner (*p* < 0.01).

**Figure 9:**
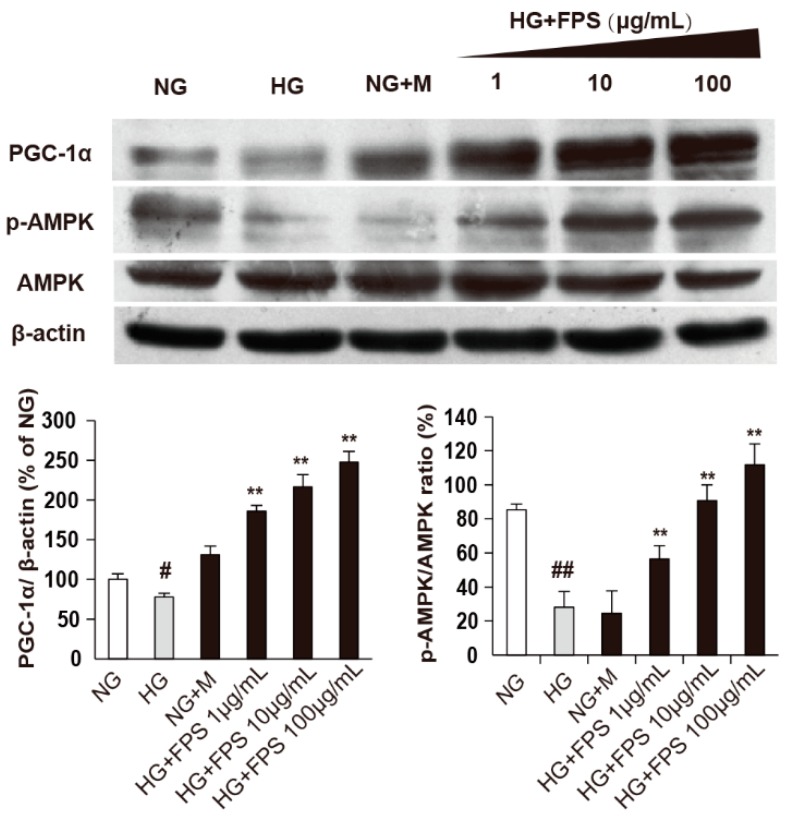
The effects of FPS on expression of AMPK and PGC-1α: (**A**) The protein expression was assayed by Western blotting; (**B**) The quantized data of protein were expressed by mean ± S.D. (*n* = 3). ## or ** *p* < 0.01; #, *p* < 0.05; “#”or “##”, HG vs. NG; “**”, HG vs. HG + FPS. The ordinate is a percentage relative to NG group.

Combining with the results above, we deduced that FPS could decrease ROS production by regulating SOD, CAT and Nox1, which was mediated by AMPK-PGC-1α pathway (Figure 9).

In this study, we used mannitol as an osmotic control. Data showed that compared to mannitol, high glucose had more significant effect on the production of intracellular ROS and protein expression levels of antioxidant enzymes. However, there was no remarkable difference in the level of activated AMPK (p-AMPK) between the high glucose group and mannitol group. This is probably because AMPK could be regulated by a variety of stimulation such as energy depletion, hypoxia, and hypertonicity [30,31].

## 3. Experimental Section

### 3.1. Materials and Reagents

*Aconitum carmichaeli* Debx was collected in July 2011 from Sichuan Province of China. The plant was identified by Dr. Peng Tan (School of Chinese Medicine, Beijing University of Chinese Medicine, Beijing, China) and voucher specimens (voucher number 20110728) were kept at materia medica Specimen Museum of this school.

d-Mannose, l-rhamnose, D-glucose were obtained from the Shanghai Yuanye Bio-Technology Co., Ltd. (Shanghai, China). Trifluoroacetic acid (TFA) and 1-Phenyl-3-methyl-5-pyrazolone (PMP) were from Shanghai Macklin Chemical Co., Ltd. (Shanghai, China). Acetonitrile (HPLC-grade) was purchased from Merck (E. Merck, Darmstadt, Germany). Other chemistry reagents were all analytical grade.

RIPA Lysis Buffer, 29% acrylamide: 1% bisacrylamide, Tris-base, glycine, TBST, ammonium persulfate (APS), tetramethylethylenediamine (TEMED), Resolving gel buffer, Stacking gel Buffer and prestained protein marker were purchased from APPLYGEN (Beijing, China). Polyvinylidene fluoride (PVDF) membrane was provided from Millipore ((Billerica, MA, USA) and all main antibodies used in this paper were obtained from Abcam (Cambridge, UK).

### 3.2. Chemistry

#### 3.2.1. Extraction of the Polysaccharides from Fuzi

The coarse powder (200 g) of Fuzi was extracted by 10 times of water under refluxing at 100 °C for 3 h. The resultant extracting solution was concentrated to 500 mL under reduced pressure. Then precipitated the polysaccharides with 2000 mL anhydrous alcohol and kept at 4 °C overnight [32]. The crude polysaccharides precipitations were obtained by centrifugation at 4000 rpm for 20 min and then the precipitate was dissolved in water again and repeated 80% alcohol-precipitation operation. After 24 h, the precipitate was re-suspended with pure water and protein was dislodged by Sevag method. The layer of water was freeze-dried after pressure reduction condensation and the Fuzi polysaccharides were obtained. The total sugar content of polysaccharides was 70.24% with method of Phenol-sulfuric acid.

#### 3.2.2. The Monosaccharide Composition Analysis of FPS

##### Acid Hydrolysis of the Polysaccharides

The polysaccharide sample (10 mg) was hydrolyzed in boiling water bath for 8 h with 2 M TFA (2 mL) [23,24]. After restoring at ambient temperature, the sample was centrifuged at 4000 rpm for 15 min. Then, the supernatant was gathered and dried under a reduced pressure.

##### Derivatization Treatment

The monosaccharides were dissolved in ammonia to prepare a solution with 1 mmol/L concentration. Then, the 450 μL standard solution was mixed with a same volume of 0.5 M methanol solution of PMP in a clean tube with stopper. The reaction occurs at 70 °C water-bath for 30 min and then cool the mixture to normal temperature and neutralize with the amount of glacial acetic acid. Then chloroform (2.0 mL) was mixed. They were shaken vigorously, and the extraction process was repeated three times. The chloroform layer was abandoned and the supernatant was centrifuged at 8000 rpm for 15 min. After filtering through 0.45 μm nylon membrane, the sample was obtained for later HPLC-MS^n^ analysis. The hydrolytic polysaccharide was dissolved in 1 mL ammonia and derived with the same processes used in monosaccharide [21,23]. In this process, the ammonia solvent without any monosaccharide was used as blank control.

##### Chromatography and Mass Spectrometry Conditions

Firstly, the analysis was performed on a Shimadzu HPLC system (Shimadzu LC-6A, Shimadzu Corporation, Kyoto, Japan). Samples were isolated with a Zorbax Extend-C18 column (250 × 4.6 mm, 5 μm, Agilent Corporation, Palo Alto, CA, USA) at room temperature. The injection volume was 20 μL with 1.0 mL/min flow rate and peaks were detected at 327 nm. The mobile phase consisted of 20 mM ammonium acetate (added 1 mL glacial acetic acid per 100 mL)–acetonitrile (80:20, *v*/*v*). Then Agilent 1100 series LC/MSD XCT plus ion-trap mass spectrometer was used for HPLC-MS^n^ analysis (Agilent, Waldbronn, Germany). Chromatographic conditions: see above. Mass spectrometer conditions were as follows: capillary voltage 3.5 kV at 350 °C; the drying gas flow rate was 11 L/min and a nebulizer pressure was 35 psi; The drying gas temperature was set at 350 °C. The MS spectrum was recorded in scanning range of *m*/*z* 100–1000.

### 3.3. Biological Effectiveness

#### 3.3.1. Cell Cultivation and Treatment Allocation

RSC96 cells, a Schwann cell line, were purchased from Cells Resource Center of Shanghai Institutes for Biological Sciences, Chinese Academy of Sciences (Shanghai, China) and incubated with Dulbecco’s modified Eagle Medium (DMEM) (Hyclone, Logan, UT, USA) containing 10% (*v*/*v*) fetal bovine serum (Gibco, UK) in the incubator with a 5% CO_2_ humidified atmosphere at 37 °C.

Treatment allocation of FPS was investigated as the following groups: 5.5 mM glucose as the normal control group (NG, normal glucose), 69.5 mM of mannitol plus 5.5 mM glucose as osmotic controls (NG + M, normal glucose + mannitol), 75 mM high glucose (HG) as model group, 75 mM glucose plus FPS with different concentrations (1, 10, and 100 μg/mL) as medicated group. Furthermore, the treatment time was 48 h.

#### 3.3.2. Detection of the Level of Active Oxygen Species (ROS)

When RSC 96 cells were cultured in high glucose condition, they often display over-production of ROS [33]. For affirming the effects of FPS on the ROS, the environment with 75 mM glucose was used to induce oxidative injury in RSC96. Then the contents of intracellular peroxide were detected by hydrogen peroxides-sensitive fluorescent probe DCFH_2_DA and superoxide anions were determined with superoxide anions-sensitive fluorescent probe DHE. Briefly, Cells (1 × 10^5^ cells/well), cultivated in 12-well plates under different culture conditions for 48 h, were collected, treated with 10 μM of DCFH_2_DA or 10 μM of DHE under 37 °C for 30 min and dark condition and then rinsed by PBS for 2 times. Images were taken by a laser scanning confocal microscope (FV1000, excitation 488 nm for DCFH_2_DA and 633 nm for DHE). The fluorescent intensity was quantified with Image J software.

#### 3.3.3. Measurement of Cell Apoptosis Rate

The equal numbers of RSC96 cells (1 × 10^6^ cells/well) were inoculated into six-well flat-bottomed plates and incubated overnight for cell adherence. Next, cells were handled with different concentrations of glucose or FPS. After 48h of treatment, the cells attached to the wall and suspended in medium were collected and washed twice with PBS. Afterwards, 1 × binding buffer was added to obtain a density of 1 × 10^6^ cells/mL and 100 μL were incubated by using the Annexin V-PE/7-AAD (Apoptosis Detection Kit from BD Biosciences, San Diego, CA, USA) double staining for 15 min under shade environment. Stained cells were analyzed by flow cytometry (DIVA) (BD FACS Canto II).

### 3.4. Mechanism

#### Determination of the Protein Expression Levels Related Oxidative Stress

About 8 × 10^5^ RSC96 cells were seeded in a 25 cm^2^ cell culture bottle and incubated overnight for cell attachment. After the same treatment with Section 3.3.3, the each group’s cells were trypsinized, rinsed three times with PBS and collected. Then the cells were added 100 μL of ice cold RIPA lysis buffer containing a protease inhibitor mixture, lysed in an ice bath for 1 h and centrifuged 10 min at 12,000 *g* at 4 °C. The protein dissolved in supernatant was acquired and concentration was determined by using the pierce BCA protein assay reagent kit (Thermo Scientific, Rockford, IL, USA).

SDS-PAGE gels with 12% Acrylamide were prepared and used to separate the mixtures of proteins sample (50 μg). The protein bands were transferred onto PVDF membrane and blocked for 1 h with 5% non-fat dry milk at 3 °C and then incubated at 4 °C over night in primary antibodies which were rabbit polyclonal anti-AMPK alpha 1 + AMPK alpha 2 antibody (ab131512, dilutions: 1:800), anti-superoxide dismutase 1 antibody (ab13498, dilutions: 1:1000), anti-NOX1 antibody (ab131088, dilutions: 1:1000), anti-catalase antibody (ab15834, dilutions: 1:1000), anti-PGC1 alpha antibody (ab54481, dilutions: 1:800), phospho-AMPKα (Thr172) (#2535, dilutions: 1:600; Cell Signaling) respectively. Subsequently, the blots were rinsed and incubated for 1.5 h at ambient temperatures with secondary antibody to Rabbit IgG-H&L (HRP) (ab16284, dilution: 1:2000). The proteins were visualized using an enhanced chemiluminescence (ECL) kit and quantified using image analysis software. In all instances, the membranes were blotted again with antibody against β-actin (dilutions: 1:1000).

### 3.5. Statistical Analysis

The quantified data were analyzed by one factor analysis of variance (ANOVA) using SPSS19.0 software (IBM Corporation, Armonk, NY, USA.). A P-value less than 0.05 was considered statistically significant.

## 4. Discussion

In traditional Chinese medicine, Fuzi, a famous herb, has been widely and successfully used for many years. In our previous study, the aqueous extract of Fuzi has been shown to ameliorate DPN in rats and improve the oxidative stress and apoptosis in Schwann cells [18]. However, the material base of Fuzi aqueous extract is unclear. Thus, in this study, we attempted to clarify the effective component of Fuzi aqueous extract. It is generally accepted that the diverse bioactivities of Fuzi predominantly originated from the diterpenoid alkaloids or polysaccharides, especially alkaloids [34]. Thus, firstly, we evaluated the antioxidant activity of the five main alkaloids of Fuzi using high glucose-stimulated Schwann cell line RSC96. However, the results showed that neoline could reduce the level of ROS without dose-dependent relationship and 14-benzoylaconine, only high concentrations, presented the antioxidant effect (data not shown). This indicated that these alkaloids may be not the main antioxidant components. Then, we extracted the polysaccharides of Fuzi (FPS) and assessed its protective potential against ROS and apoptosis. The findings indicated that FPS diminished the peroxide and superoxide anion levels in dose-dependent manner to some extent and lowered the apoptotic ratio. Meanwhile, the MS analysis results demonstrated that FPS was composed of mannose, glucose, rhamnose, galactose and so on. The previous and present studies indicate altogether that Fuzi aqueous extract may be used to improve DPN and polysaccharides are probably the effective component of Fuzi aqueous extract. 

In addition, we investigated the underlying molecular mechanisms of FPS against the oxidative stress. SOD and CAT play important roles in protecting the cell from oxidative damage by ROS [26]. SOD catalyzes the dismutation of the superoxide radical into oxygen and hydrogen peroxide. CAT catalyzes the decomposition of hydrogen peroxide to water and oxygen [26]. In contrast, Nox1 is a member of Nox family, which transfers electrons from to oxygen to produce superoxide anion [35]. AMPK is a metabolic master switch in regulating cellular energy homeostasis and AMPK activation is currently considered an important component of cellular responses to stresses that threaten cell viability, where the common one is oxidative stress [30,31,36,37]. However, AMPK activators could increase cell survival, such as metformin and dithiolethiones [38,39]. Moreover, owing to the unified pathological mechanism of diabetic complications, oxidative stress, AMPK signaling pathways has been as a novel therapeutic approach for antidiabetic treatment [40]. PGC-1α is involved not only mitochondrial biogenesis, but also ROS metabolism by regulating the transcription of a group of genes involved in ROS detoxification, such as superoxide dismutase and uncoupling protein [41]. Recently, studies have shown that AMPK can promote PGC-1α transcription [42]. Thus, in preliminarily exploring the FPS effects, the AMPK-PGC-1-alpha pathway was more particularly considered. Some other downstream targets could also be activated and function protectively in SCs, which need further exploration. 

## 5. Conclusions

In summary, combining the phytochemical and pharmacological studies, our study was a comprehensive evaluation of FPS. FPS, which includes at least nine different monosaccharides and consists of vast glucose, could ameliorate high glucose-induced oxidative injury and these benefits might work through AMPK-PGC-1α pathway.

## Figures and Tables

**Table 1:** Identification of monosaccharide composition of FPS.

No.	t_R_ (min)	MS	MS^2^	MS^3^	Mw	Saccharides
1	9	508.2	334.1	213.8	180	Unknown (C_6_H_12_O_6_)
2	10.8	509	335.1	214.9	180	Mannose (C_6_H_12_O_6_)
3	15.8	493.2	319	214.8	164	Rhamnose (C_6_H_12_O_5_)
4	24.9	509.2	335	214.7	180	Glucose (C_6_H_12_O_6_)
5	27.4	509.3	335	214.8	180	Galactose (C_6_H_12_O_6_)
6	29.4	509.3	335.1	214.7	180	Unknown (C_6_H_12_O_6_)
7	31	479.2	305	214.7	150	Xylose (C_5_H_10_O_5_)
8	31.9	479.2	305	214.8	150	Arabinose (C_5_H_10_O_5_)
9	38.5	493.3	319.1	214.7	164	Fucose (C_6_H_12_O_5_)
